# Antiphospholipid antibodies in patients with antiphospholipid syndrome

**DOI:** 10.11613/BM.2024.020504

**Published:** 2024-06-15

**Authors:** Slavica Dodig, Ivana Čepelak

**Affiliations:** Department of Medical Biochemistry and Hematology, Faculty of Pharmacy and Biochemistry, University of Zagreb, Zagreb, Croatia

**Keywords:** antiphospholipid syndrome, antiphospholipid antibodies, lupus anticoagulant, anti-β2-glycoprotein I, anticardiolipin antibodies

## Abstract

Antiphospholipid syndrome (APS) is a rare systemic autoimmune disease characterized by recurrent pregnancy morbidity or thrombosis in combination with the persistent presence of antiphospholipid antibodies (aPLs) in plasma/serum. Antiphospholipid antibodies are a heterogeneous, overlapping group of autoantibodies, of which anti-β2-glycoprotein I (aβ2GPI), anticardiolipin (aCL) antibodies and antibodies that prolong plasma clotting time in tests *in vitro* known as lupus anticoagulant (LAC) are included in the laboratory criteria for the diagnosis of APS. The presence of LAC antibodies in plasma is indirectly determined by measuring the length of coagulation in two tests - activated partial thromboplastin time (aPTT) and diluted Russell’s viper venom time (dRVVT). The concentration of aβ2GPI and aCL (immunglobulin G (IgG) and immunoglobulin M (IgM) isotypes) in serum is directly determined by solid-phase immunoassays, either by enzyme-linked immunosorbent assay (ELISA), fluoroimmunoassay (FIA), immunochemiluminescence (CLIA) or multiplex flow immunoassay (MFIA). For patient safety, it is extremely important to control all three phases of laboratory testing, *i.e.* preanalytical, analytical and postanalytical phase. Specialists in laboratory medicine must be aware of interferences in all three phases of laboratory testing, in order to minimize these interferences. The aim of this review was to show the current pathophysiological aspects of APS, the importance of determining aPLs-a in plasma/serum, with an emphasis on possible interferences that should be taken into account when interpreting laboratory findings.

## Graphical abstract

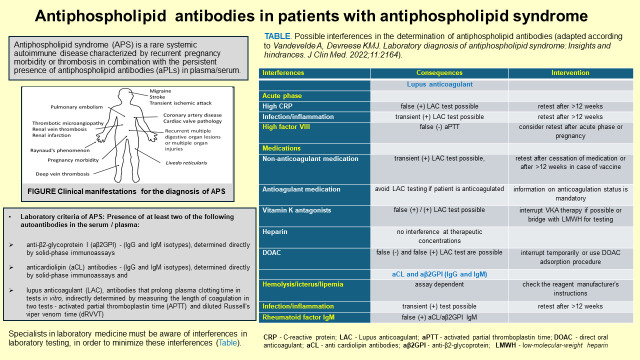

## Highlights

• Antiphospholipid syndrome is a rare systemic autoimmune disease characterized by recurrent pregnancy morbidity or thrombosis in combination with the persistent presence of antiphospholipid antibodies in plasma/serum

• Specialists in laboratory medicine should take responsibility for the entire analytical process, so that possible interferences are minimized, and physicians obtain reliable results of the patient’s laboratory findings in a timely manner

• Due to possible problems in performing tests on aPLs for a more reliable (optimal) interpretation of laboratory findings, a close cooperation between laboratory specialists and clinical specialists is needed

## Introduction

**Figure 1:**
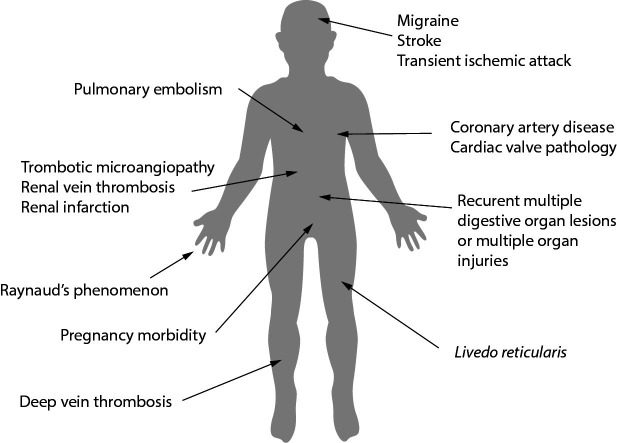
Manifestations of antiphospholipid syndrome.

Antiphospholipid syndrome (APS) is a rare systemic autoimmune disorder characterized by heterogeneity in the clinical spectrum that includes arterial, venous or microvascular thrombosis, pregnancy morbidity (recurrent miscarriages, premature births and preeclampsia) or non-thrombotic manifestations, in combination with the persistent presence of autoantibodies, *i.e.* antiphospholipid antibodies (aPLs) respectively (*1*-*3*). According to some epidemiological studies, annual incidence is estimated to be between 1 and 2/100,000 and the prevalence is between 40 and 50/100,000 (*4*). Most patients with APS are diagnosed between the ages of 15 and 50, more often in women. In the elderly, APS appears after the age of 50 in less than 13% of individuals, more often in men. The most common manifestations of APS are miscarriage or fetal loss, thromboembolism, thrombosis, heart attack or transitory ischemic attack, Raynaud’s phenomenon/syndrome, catastrophic APS (≥ three organs affected), *etc.* (Figure 1). So far there is no answer to the question why some aPL carriers never develop any of the APS manifestation.

Antiphospholipid syndrome can be classified as both primary and secondary disorder, respectively. Patients with primary APS have no clinical or laboratory evidence of another disease. Secondary APS can appear in combination with other diseases, either autoimmune diseases (most usually with systemic lupus erythematosus), infections which induce the production of aPL’s (human immunodeficiency virus (HIV), *varicella-zoster* virus, hepatitis C virus, infections of skin, urinary tract, respiratory tract), cancer (hematological malignancies and solid tumours) or with the use of some medicatons such as chlorpromazine, phenytoin, hydralazine, procainamide, quinidine, amoxicillin, chlorothiazide, propranolol, oral contraceptives, *etc.* (*5*, *6*). Infections, sepsis, malignancies or medications are the triggering or risk factors (known as second hit) that are required in combination with aPLs to trigger a thrombotic event.

Antiphospholipid antibodies constitute a heterogenous family of antibodies directed mainly against negatively charged (anionic) phospholipides or plasma phospholipid-binding proteins (*7*, *8*). Among these antibodies lupus anticoagulant (LAC), anticardiolipin antibodies immunoglobulin M/immunoglobulin G (aCL IgM/IgG), and IgM/IgG anti-β2-glycoprotein I (aβ2GPI IgM/IgG) are crucial for diagnosing APS. These antibodies are included in the laboratory classification criteria for the diagnosis of APS (*1*). Apart from classification criteria aPLs, other antibodies, known as non-criteria aPLs, can be found in the patient’s serum.

Seronegative antiphospholipid syndrome is diagnosed in patients who have clinical manifestations of APS, but do not meet the laboratory criteria, because they do not have detectable aPLs values in plasma/serum, which may indicate a possible insufficient analytical sensitivity of immunoassays (*9*-*11*).

The introduction of the enzyme-linked immunosorbent assay (ELISA) method made the determination of autoantibodies aCL more accessible in everyday laboratory practice (*12*, *13*). Determination of anti-β2GPI antibodies were introduced in 2006 (*14*). Alternative methods to ELISA such as automated fluorescence enzyme immunoassay (FEIA) and chemiluminescence immunoassay (CLIA) were introduced recently (*15*, *16*).

With this review, we wanted to show the current pathophysiological aspects of APS, the importance of determining aPLs in plasma/serum, with an emphasis on possible interferences, especially preanalytical ones, which should be taken into account when interpreting laboratory findings.

For the purpose of writing this review, the literature search was done from January 2010 to January 2024, focusing more on the latest research. The PubMed, Medscape, ResearchGate, National Library of Medicine and Academia.edu databases were used with the medical subject heading key words “antiphospholipid syndrome”, “patomechanism“, “antiphospholipid antibodies”, “lupus anticoagulant”, „laboratory investigations“, „biomarkers“, review“. In addition, articles from the reference sections of selected manuscripts were included.

## Proposed pathogenesis of antiphospholipid syndrome

The pathomechanism of APS is mainly viewed from three aspects - through thrombotic mechanism, obstetric mechanism and immune mechanism, which mainly include endothelial cell activation, platelet activation, thrombosis, inflammation, complement activation. The exact triggers for the production of aPLs in APS are not fully understood, but both genetic predisposition and environmental factors, such as infections and medications, may play a role (*3*, *5*).

### Thrombotic mechanism

**Figure 2:**
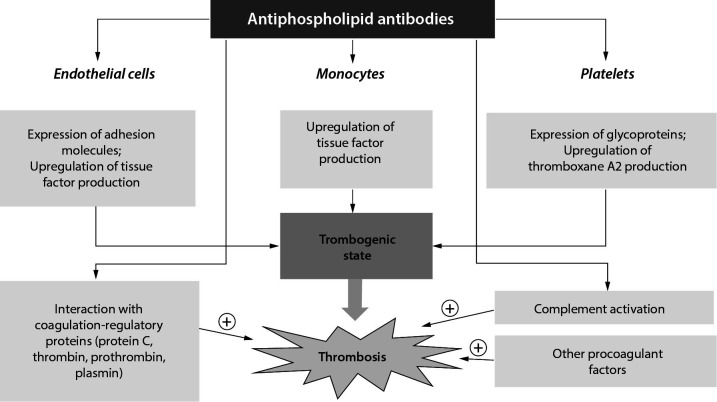
Procoagulant mechanisms of antiphospholipid antibodies (*21*).

It is proposed that procoagulant mechanisms of aPL (Figure 2) are mainly mediated by antibody reactivity against phospholipid-binding proteins on membranes of different cells, such as endothelial cells, monocytes, platelets and neutrophils (*17*-*20*). This is followed by the expression of adhesion molecules (on endothelial cells), glycoproteins (on platelets) and upregulation of tissue factor production, which leads to a thrombogenic state. However, it seems that these processes are not sufficient for thrombosis to occur, but activation of the complement cascade by aPL (C3a, C5a and the C5b-9 complex, *i.e.* membrane attack complex, MAC) is required. At the same time, aPLs induce interaction with coagulation-regulatory proteins (protein C, thrombin, protrombin, plasmin) and the inclusion of other procoagulant factors (factors VIIa, IXa, and Xa), which additionally leads to thrombosis (*21*). The presence of aPLs is probably an independent risk factor for atherosclerosis, most likely through binding to β2GPI found in numerous lipoprotein fractions, including oxidized low density lipoprotein cholesterol (oxLDL) (*17*).

### Obstetric mechanism

Pregnancy morbidity may occur in women with APS, which includes some clinical criteria, such as: (i) ≥ 3 unexplained consecutive miscarriages before 10th week of gestation, with maternal anatomic or hormonal abnormalities and paternal and maternal chromosomal causes excluded; or (ii) ≥ 1 unexplained fetal losses of a morphologically normal fetus beyond the 10th gestational week; or (iii) stillbirth or prematurity due to eclampsia or severe preeclampsia or placental insufficiency before the 34th week of gestation, due to extensive placental thrombosis and compromised fetal circulation (*1*, *22*). In the pathogenesis of pregnancy morbidity, aβ2GPI autoantibodies play an important role, which, after the initial activation of proinflammatoy factors, *eg*. neutrophils, interleukin 8 (IL-8), tumor necrosis factor alpha (TNF-α), induce oxidative stress, thrombosis and activation of the complement system (C3 and C5). Components C4a and C3b are deposited in the placenta and the concentration of C3 and C4 in the serum decreases. The most likely mechanism by which aPLs cause spontaneous abortion is interaction with placental annexin V and activation of complement. Placental dysfunction leads to impaired placental perfusion, resulting in fetal death.

### Immune mechanism

Immune mechanisms in APS include immune complex deposition, immune cross-reaction, interference by LAC, the production of neoantigens in cell membrane rupture and the direct action of aPLs. Immunoglobulin deposition was confirmed in the heart valves in APS patients with valvular heart diseases, also in the gastrointestinal tract, which suggests that aPLs may be involved in the inflammatory process through interaction with tissue surface antigens. In addition, it was shown that aCLs can cross-react with liver mitochondria (*23*).

A study by Yan *et al.* has shown that in patients with APS there is an imbalance in lymphocyte and T cell subsets. In comparison with the control group, patients with APS had increased number of Th1 cells and decreased number of Th2 cells, decreased number of Treg cells, increase Th17/Treg ratio. In addition, patients with primary APS had a significant decrease in the number of NK cells, and patients with secondary APS had decreased values of T, B and CD4+T cells (*24*).

## Clasification criteria of antiphospholipid syndrome

The first classification criteria of APS, mainly related to the clinical characteristics of the disorder, were accepted at the 8th International Congress in Japan in 1998 (*14*). These criteria were updated several times and included both, clinical (vascular thrombosis and pregnancy morbidity) and laboratory criteria (*e.g*. aβ2GPI), respectively. The last revision was recommended by American College of Rheumatology (ACR) and European Alliance of Associations for Rheumatology (EULAR) and published in October 2023 (*1*).

The diagnosis of APS typically requires the presence of both clinical criteria and laboratory evidence of antiphospholipid antibodies. The clinical criteria for the diagnosis of APS, which were developed by the use of rigorous methodology with multidisciplinary international input, are classified into the following domains: macrovascular arterial thrombosis, macrovascular venous thromboembolism, microvascular, obstetric and cardiac valve (*1*).

A definitive diagnosis of APS is established if at least one clinical and one laboratory criteria are met, with the first measurement of the laboratory test performed at least 12 weeks from the clinical manifestation. The latest classification criteria for the diagnosis of APS, include the following laboratory criteria: (i) persistent positive LAC, (ii) persistent aPLs, *i.e*. aCL and anti-β2GPI antibodies (IgG/IgM isotypes), moderate or high positive (IgM alone) (aCL and/or anti-β2GPI), moderate positive (IgG) (aCL and/or anti-β2GPI) with or without IgM, high positive (IgG) (aCL or anti-β2GPI) with or without IgM, high positive (IgG) (aCL and anti-β2GPI) with or without IgM (*1*).

Additional clinical and laboratory criteria imply the determination of scores for particular clinical and laboratory findings. The severity of aPLs findings can be assessed using single *vs* double *vs* triple aPL positivity for APS. Single positive LAC and moderate or high positive aCL and/or aβ2GPI/IgM antibodies (1 point) have the lowest risk, followed by moderate positive aCL and/or aβ2GPI/IgG antibodies (4 points), then persistent positive LAC and high positive aCL or aβ2GPI /IgG antibodies (5 points) and finally high positive aCL and aβ2GPI/IgG antibodies (7 points) (*25*). At the same time, it is important to know whether aβ2GPI and aCL were determined using standardized ELISA or new immunochemical assays on automated analyzers. Namely, the cut-off values on the basis of which the positivity or negativity of the findings are determined differ between these methods.

Due to the loss of peripheral immune tolerance in patients with APS, the synthesis of various aPLs occurs. For many years it was assumed that aPLs are directed against native, “self” phospholipids. However, Hörkkö and Grygiel-Górniak have shown that most aPLs (*e.g*. aCL) are directed toward epitopes of oxidized phospholipids (*26*, *27*). As already mentioned, the laboratory criteria for the diagnosis of APS include antibodies LAC, aCL and aß2PLI, mainly IgG, IgM classes. Unlike IgG and IgM aCL/aβ2GPI, the role of IgA aCL and IgA aβ2GPI in APS is not yet clear, so IgA antibodies are not included in the current laboratory criteria of APS. After aβ2GPI binds to its antigen, the conformation of β2GPI changes. The closed conformation of β2GPI transforms into an open conformation (*28*). Binding of antibodies to target protein-binding proteins changes their basic functions. The formation of complexes between CL and β2GPI bound to proteins with their specific antibodies (aCL and aβ2GPI) can result in a change in anticoagulant processes or in an increase in procoagulant effects (*8*).

## Features of β2-glycoprotein I and cardiolipin

### β2-glycoprotein I

Beta-2-glycoprotein I protein, also known as apolipoprotein H (apoH) is a soluble plasma protein, relative molecular weight (Mr), of 48 kDa, contains 326 aminoacids organized into five domains. It is expressed on the surfaces of several cells, *e.g.* trophoblasts, endothelial cells, monocytes and platelets (*28*). It is known that β2GPI exists in at least two different conformations, a closed and an open conformation (90% of the β2GPI circulates in the blood and has a closed conformation).

**Figure 3:**
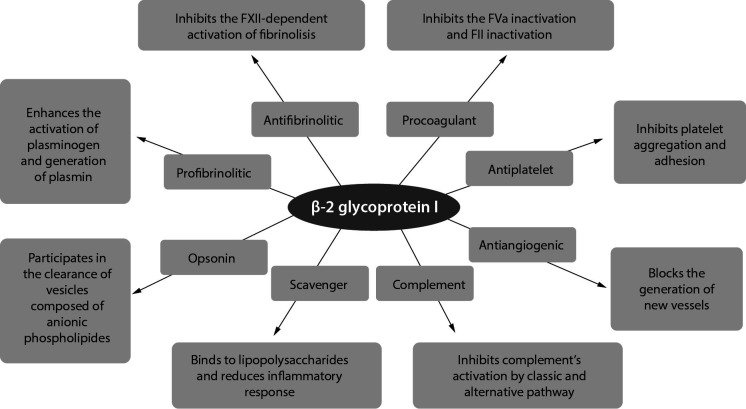
Functions of β2glycoprotein I.

Beta-2-glycoprotein I protein is a unique protein with a key role in hemostasis and immunity (Figure 3). Scientific studies have shown that β2GPI may bind to several biological molecules that participate in coagulation and the complement system (*e.g.* C3 component, glycoprotein 1b, annexin A2, plasminogen, fibrin, factor XII, von Willebrand factor and platelet factor 4). In the process of hemostasis, it has anticoagulant and antithrombotic, but also procoagulant effects, which can be direct or indirect. Beta-2-glycoprotein I protein directly affects coagulation by inhibiting the thrombomodulin complex (procoagulant) and binding thrombin to reduce its activity (anticoagulant); also, it regulates platelet activation (*29*). Indirectly, β2GPI has an anticoagulant effect through the regulation of thrombin generation; its indirect coagulation effect is manifested through the inhibition of protein C activation and interruption of the anticoagulant annexin V shield. Whether anticoagulant or procoagulant effects will prevail depends on the surrounding environment. In addition, β2GPI activates platelets and promotes clot formation. How β2GPI affects the complement system is not clear. Four domains of β2GPI are known to be similar to complement control protein (CCP), so some authors hypothesize that β2GPI acts as a cofactor of complement inhibition (*30*). Beta-2-glycoprotein I protein can reduce the inflammatory response through two processes: removal of lipopolisaccharide (LPS) and opsonization that ultimately leads to the removal of vesicles composed of anionic phospholipids (*5*).

### Cardiolipin

**Figure 4:**
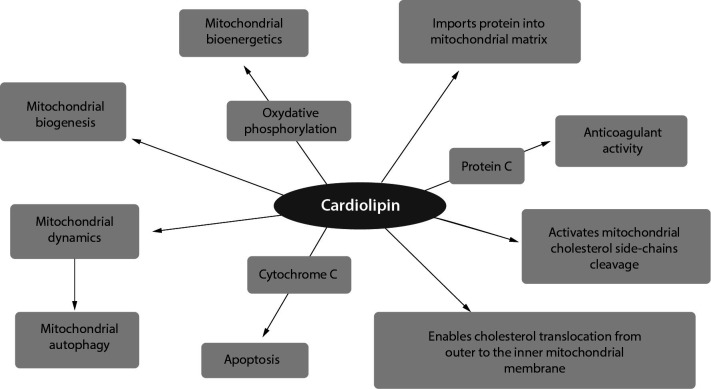
Functions of cardiolipin.

Cardiolipin, CL (1,3-bis(*sn*-3’-phosphatidyl)-sn-glycerol) is a phospholipid (diphosphatidylglicerol) with unique structure that consists of two phosphate residues and four types of fatty acyl chains (*31*). It is exclusively synthesized and localized in the inner mitochondrial membrane, where it makes up about 20% of the total lipid composition. Cardiolipin participates in numerous reactions involved in mitochondrial bioenergetics, especially the process of the oxidative phosphorylation. Cardiolipin is also considered to participate in mitochondrial autophagy (mitophagy) and to trigger apoptosis. It plays an important role in regulation of mitochondrial proteins, *e.g.* carrier proteins and phosphate kinases, electron transport complexes, cholesterol translocation from outer to the inner mitochondrial membrane, activates mitochondrial cholesterol side-chain cleavage, import protein into mitochondrial matrix and enhances protein C pathway anticoagulant activity (Figure 4). In this way, CL plays a central role in many reactions and processes important for maintaining mitochondrial function (*10*, *32*).

## Antiphospholipid antibodies - laboratory markers of antiphospholipid syndrome

### Lupus anticoagulant

According to the current laboratory criteria, to establish a diagnosis of APS, it is necessary to confirm the permanent presence of aPLs in the serum. Moreover, as the serum of individual patients with APS contains various types of aPLs with different specificities, there is no single laboratory test that can diagnose APS, much less a single biomarker that can be considered the diagnostic gold standard for APS (*33*). It is considered that diagnostic aPLs can be generally categorized into two groups, *i.e.* as antibodies whose presence is indirectly proven by their ability to prolong coagulation tests dependent on phospholipids (*e.g.* primarily LAC) and antibodies that may be detected by solid-phase immunoassays (*e.g.* primarily aCL, aβ2GPI) (*20*).

The main disadvantages of aPL testing are that there is no standardization of methods, there are no prognostic biomarkers for APS and it is not known whether aPL tests detect exactly those autoantibodies that would be responsible for the clinical manifestations of APS (*34*, *35*).

Lupus anticoagulant, also referred to as nonspecific inhibitors that block phospholipid surfaces important for coagulation, is a term that includes aPLs. Still, it is important to point out that the term LAC does not refer to a single antibody, since it includes at least three types of antibodies - aCLs, aβ2GPI and antiprothrombin antibodies, predominantly IgG, and IgM isotypes (*36*). Antibodies against β2GPI and prothrombin have the most significant association with pathogenicity in patients with APS. Several studies have shown that triple positivity (all three antibodies) correlates more strongly with both thrombosis and pregnancy morbidity than the presence of double or single positivity (*37*).

Lupus anticoagulant is detected using functional, phospholipid-dependent coagulation assays. It cannot be measured directly, but is determined using a panel of sequential tests, including diluted Russell’s viper venom time (dRVVT, based on the ability of the snake’s venom to accelerate blood clotting) and activated partial thromboplastin time (aPTT, a test to detect disorders of the intrinsic and common coagulation pathway) assays, which are tested three times (*38*). In the dRVVT test, the enzyme from Russell’s viper venom directly activates factor X. In the patient’s plasma aPLs will react with the phospholipid components, which will result in a prolongation of dRVVT and a decrease in the activity of the prothrombin activator complex (*38*). The principle of aPTT is based on the activation of the contact (intrinsic) coagulation pathway. In a patient with APS, aPLs present in plasma inhibit phospholipid-dependent steps in the aPTT coagulation pathway.

**Figure 5:**
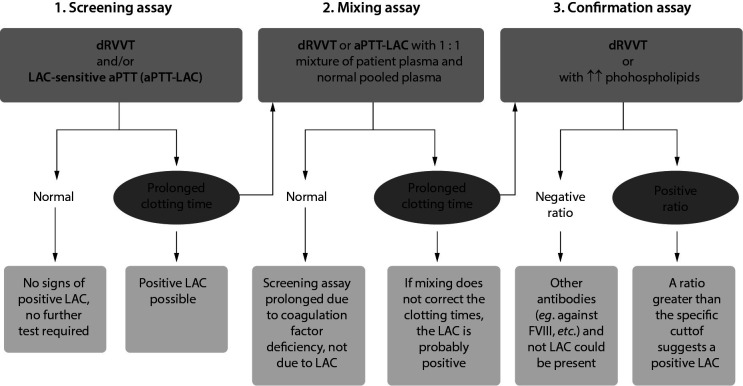
Lupus anticoagulant (LAC) determination algorithm (*38*). The ratio is obtained by dividing the result with a low phospholipid concentration by the test with a high phospholipid concentration. dRVVT - dilute Rusell viper venom test. aPTT - activated partial thromboplastin time.

As the patient’s platelet-poor citrated plasma is taken for LAC determination, the blood sample should be drawn into 0.109 M sodium citrate blood collection tubes (the blood-to-anticoagulant ratio of 9:1). Some recommendations for the optimal laboratory detection of LAC require the following: (i) samples should be transported to the laboratory at room temperature; (ii) double centrifugation is required; (iii) the sample should be processed within four hours of collection. If this is not possible, the sample must be aliquoted and frozen (- 20 to - 80 °C) until analysis; (iv) three tests are used to detect LAC. The initial determination begins with a screening test (LAC-screen) that determines dRVVT and aPTT in patient’s plasma, followed by a test that confirms or excludes the presence of LAC (Figure 5) (*39*, *40*). This is achieved by analyzing a mixed sample consisting of equal volumes of patient’s plasma with “normal pooled plasma“ (1:1). In this mixed sample, dRVVT and aPTT are determined. The next step is the confirmatory test (LAC-confirm), where excess phospholipids are added to the patient’s sample and dRVVT and aPTT are determined. The results are then compared with the LAC-screen, followed by the interpretation of results, and that are expressed as positive or negative. The aPTT assay is more sensitive, and dRVVT is more specific for LAC (with the note that both tests depend on the reagents used). If the patient is taking vitamin K antagonists, predilution of the sample with „normal pooled plasma“ testing is not recommended since it also leads to dilution of the LAC activity by 50% (*41*).

The strength of the LAC is expressed as a ratio between screening assay and confirmation assay, preferably normalized on the mean of the normal population, according to the following equation: normalized LAC ratio = (Screen (patient)/Screen (normal)) / (Confirm (patient)/Confirm (normal)) (*33*).

If LAC is determined in patients taking vitamin K antagonists, it is necessary that the value of the international normalized ratio (INR) is within the therapeutic range (INR = 2-3); if INR values are > 3, determination of LAC is not recommended. Compared with directly oral anticoagulant drugs, DOACs (such as dabigatran, rivaroxaban, apixaban, and edoxaban), vitamin K antagonists remain the standard of care in the treatment of APS (*33*). Particular caution should be exercised if INR is determined on point-of-care testing (POCT) devices. Noordermeer *et al.* have shown that LAC can interfere with INR results in POCT, which depends on the potency of the used thromboplastin reagent (*42*). In patients with APS who are taking vitamin K antagonists and have increased values of aCL, aβ2GPI and LAC, it is not recommended to monitor the value of the INR determined by the POCT device, especially if the INR is > 3.0.

Research has shown that various interferences can occur when determining LAC, which can result in either false positive or false negative test results. For example, falsely increased values are obtained in patients with acute inflammation who have increased values of C-reactive protein, because it shows affinity to phospholipids, thereby interfering with aPTT testing and leading to a false positive result of the LAC test (*43*). Falsely reduced values (the clotting time of aPTT is reduced) occur in patients with increased coagulant activity of factor VIII (*e.g.* during pregnancy, inflammation, acute thrombotic event, after surgery, in malignant diseases, *etc.*). Concentration of FVIII does not affect dRVVT screening because factor X is directly activated by Russell’s snake venom. Unreliable results are also obtained with the use of some medications, primarily with anticoagulants, *e.g.* vitamin K antagonists and DOACs (*44*, *45*). Certainly, when determining the LAC, these situations should be taken into account. Re-testing of LAC after 12 weeks increases the reliability of this laboratory method (*7*).

### Anti β2 glicoprotein and anticardiolipin antibodies (IgG/IgM) - principle of determination

Both types of antibodies (aβ2GPI and aCL) are determined by solid-phase immunoassays, either by ELISA, FIA or CLIA methods, including multiplex immunoassays (*8*, *46*-*48*).

Before determining the concentration of aCL, it is necessary to dilute the serum (1:100) to remove rheumatoid factor and human immunoglobulins interference (*e.g*, when determining the IgM isotype, the influence of IgG should be reduced). Samples can be stored at 2-8 °C for up to seven days or stored at - 20 °C for up to six months.

Although a positive finding of LAC is considered a key predictor of the clinical manifestation of APS, according to current criteria aβ2GPI and aCL IgG/IgM antibodies have the same value. In immunoassays, for the determination of aβ2GPI antibodies, β2GPI is used as an antigen; cardiolipin and β2GPI are used to determine aCL. Detection of aβ2GPI antibodies is challenging, as some antibodies may be directed against a cryptic epitope that is exposed only after a conformational change in shape. The exposure of this cryptic epitope in the first domain of β2GPI varies in different commercial aβ2GPI IgG assays (*49*). Traditionally, APLs are determined by the ELISA method. Today, other immunochemical methods are in use on automated analyzers, which use different carriers (*e.g.* magnetic beads) and different types of detection (fluorescent or chemiluminescent compounds). So far, there is no consensus on the “gold standard” for the determination of APLs in serum. As different laboratories apply different methods from different manufacturers, external quality control is almost impossible. For the longitudinal monitoring of a patient with APS, determination of the concentration of aPLs should always be performed using the same method. It is necessary to know the limit of detection (functional sensitivity) and to determine both, inter-assay and intra-assay precision of each method.

As immunochemical methods developed, so did the methods for determining aPLs - from ELISA, FIA (*50*-*54*). The basic principle of all mentioned immunoassays is the same - concisely, the solid-phase (microparticles) are coated with antigens (β2GPI or CL); patient’s serum (possibly containing specific antibodies) is added; after incubation and washing, a reagent containing anti-human IgG or IgM antibodies linked to a conjugate that can bind to the Fc portion of the patient’s antibody is added. Depending on the indicator (enzyme, fluorescent compound or chemiluminescent compound) the final signal of the reaction (color, fluorescence or chemiluminescence) is developed, which is measured by a suitable detector (spectrophotometer, fluorometer, luminometer). This principle is characteristic of labeled immunochemical methods (*55*). By comparing the signal with the calibration curve, the antibody concentration is quantified.

As there is still no international reference standard for aβ2GPI and aCL, the results are expressed in relative, arbitrary units and not in international units. The results obtained by the ELISA method for both, aβ2GPI and aCL are expressed in standard IgM units (SMU) and standard IgG units (SGU) based on the calibration curve, which typically refers to the relative concentration the autoantibodies being measured. The standardization of units for FIA, CLIA and MFIA, can vary depending on the specific assay kit, manufacturer and analyzer. The units “U/mL” are mainly used to express the concentration of autoantibodies. However, the specific definition of one unit may vary between assays. Some manufacturers may calibrate their assay kits using reference materials or calibrators to establish standardized units, where one unit represents a defined concentration or activity of the target autoantibodies. This standardization may not be consistent across all assay kits or laboratories. In any case, the interpretation of results relies on comparison to internal laboratory cut-off values. In addition, FIA and CLIA are more sensitive than ELISA and are used to determine very low concentrations of autoantibodies (*56*).

### Cut-off values of antiphospholipid antibodies

The value of aPLs depends mainly on the selected reference population, the characteristics of the applied solid-phase immunoassay, the statistical method used to determine the threshold values, and the sensitivity and specificity of the defined cut-off values. Vanoverschelde *et al.* determined the cut-off values of aPLs with four immunochemical methods: ELISA, FIA, CLIA and MFIA, and results are presented in Table 1 (*46*). According to the new criteria as a moderate to high and high level threshold for aCL and ab2GPI are given as 40-79 and > 80 units based on ELISA, respectively (*1*).

**Table 1:** Manufacturer’s 99th cut-off values of aβ2GPI and aCL (both IgG and IgM isotypes) determined with different immunochemical methods - ELISA, FIA, MFIA and CLIA*

**Antibody**	**ELISA** **(SMU, SGU)**	**FIA (U/mL)**	**MFIA (U/mL)**	**CLIA (U/mL)**
aβ2GPI (IgM / IgG)	20	10	20	20
aCL (IgM / IgG)	20	10	20	20
*It is important to note that cut-off values should be regarded as recommendations only. Each laboratory should establish its own cut-offs. ELISA - enzyme-linked-immunosorbent assay. FIA - fluorescence enzyme immunoassy. MFIA - multiplex flow immunoassay. CLIA - chemiluminiscent immunoassay. aβ2GPI - anti-β2-glycoprotein I antibodies. aCL - anticardiolipin antibodies. SMU - standard IgM units. SGU - standard IgG units.				

Vanoverschelde *et al.* found that the values of aPLs in healthy subjects were not normally distributed and therefore the cut-off values were calculated using the nonparametric method (*46*). Based on the determination of the 95th and 99th percentiles, they concluded that the 99th percentiles correlated better with thrombosis, reduced sensitivity, but at the same time increased specificity (reduce the number of false positive results). So, the results greater than the 99th percentiles should be considered positive. The authors also showed that the sensitivity and specificity in healthy individuals differ from the values declared by the test manufacturers, indicating that each laboratory should create own cut-off values for aPLs (Table 2). There is still a need to define standardized statistical criteria according to which the 99th percentile cut-off reference values would be calculated.

**Table 2:** Sensitivity and specificity for 99th percentile cut-off values of antiphospholipid antibodies according to manufactures data and healthy volunteers

**Antibody**	**Sensitivity (%)**	**Specificity (%)**
	**Manufacter’s data**	
aβ2GPI (IgM)	7.1-9.9	94.7-96.3
aβ2GPI (IgG)	8.7-16.3	88.5-96.6
aCL (IgM)	16.3-26.2	96.0-99.4
aCL (IgG)	16.7-22.2	96.9-99.1
	**Healthy volunteers**	
aβ2GPI (IgM)	4.0-13.9	91.3-97.8
aβ2GPI (IgG)	14.3-23.0	95.3-99.7
aCL (IgM)	0.8-15.9	88.8-99.1
aCL (IgG)	10.7-23.0	95.0-100.0
aβ2GPI - anti-β2-glycoprotein I antibodies. aCL - anticardiolipin antibodies. IgM - immunoglobulin M. IgG - immunoglobulin G.		

After the diagnosis of APS and confirmed positive aPLs, the values of antiphospholipid antibodies can be monitored longitudinally, depending on the clinical manifestations of the disease. It was shown that more than half of patients with initial medium-high aPL values had persistently positive aPL over time. With multiple aPL positivity, the odds of a persistent aPL profile increased. If patients had isolated positive values of LAC and aPLs, the positivity decreased (*57*).

Due to the high variability among these methods, it is recommended that β2GPI or CL (IgM/IgG) antibodies are always determined by the same methods. It is also important, as with all immunochemical methods, to monitor the values of these specific antibodies longitudinally, using the same methods, preferably in the same laboratory. In cases where the results of determining the concentration of aβ2GPI and aCL are not proven in patients with a strong clinical suspicion of APS, serum analysis can be repeated in another laboratory that applies another test platform. In daily practice, in-house cut-off values for each method are applied, which were obtained by analyzing a sufficiently large population, *i.e.* at least 120 of healthy reference individuals (*58*).

### Non-criteria antiphospholipid autoantibodies

Today, more than 30 non-criteria aPLs are known. Among them are “first-line” aPLs such as: antibodies against phosphatidylserine/prothrombin complex (aPS/PT IgG/IgA/IgM), aβ2GPI Domain I, IgA of aβ2GPI and aCL, which are highly specific for the identification of APS patients (*9*). Some non-criteria aPLs can be applied as potential biomarkers to predict the risk of thrombosis in APS, such as anti-phosphatidylserine/prothrombin complex antibodies (aPS/PT IgG/IgA/IgM), phosphatidylserine antibodies (aPS IgG) and antibodies directed against phospholipids, anexin V (*17*, *59*, *60*). These antibodies are included in the laboratory classification criteria for the diagnosis of APS. According to some authors, non-criteria aPLs can increase the diagnostic value for APS. In addition, they can contribute to better recognition of seronegative APS (*9*). However, according to available data, the added value of these antibodies is still not clear enough to include them in routine practice. In the future, multicenter studies are needed to determine how much non-criteria aPLs could improve the diagnostic value in APS.

### Preanalytical considerations and interferences

Interferences can arise due to the presence of rheumatoid factor, increased concentrations of immunoglobulins and the presence of heterophilic antibodies in the serum (*61*). Although more and more attention is paid to interferences today, it is likely that some interferences will still remain unrecognized. Perhaps the possibility of adding information about known interferences to the patient’s report could be considered. Reagent manufacturers specify possible interferences in the packing instructions, such as hemolysis/icterus/lipemia or the presence of rheumatoid factor in the patient’s serum. The presence of IgM rheumatoid factor can cause false positive results of aCL (IgM) and aβ2GPI (IgM). Transient increases in aCL and aβ2GPI are present in patients with infectious diseases and other inflammatory conditions, again emphasizing the importance of repeat aPL testing after more than 12 weeks (Table 3).

**Table 3:** Possible interferences in the determination of antiphospholipid antibodies

**Interferences**	**Consequences**	**Intervention**
	**Lupus anticoagulant**	
**Acute phase**		
High CRP	False (+) LAC test possible	Retest after > 12 weeks
Infection/inflammation	Transient (+) LAC test possible	Retest after > 12 weeks
**High factor VIII**	False (-) aPTT	Consider retest after acute phase or pregnancy
**Medications**		
Non-anticoagulant medication	Transient (+) LAC test possible	Retest after cessation of medication or after > 12 weeks in case of vaccine
Antioagulant medication	Avoid LAC testing if patient is anticoagulated	Information on anticoagulation status is mandatory
Vitamin K antagonists	False (+) / (+) LAC test possible	Interrupt VKA therapy if possible or bridge with LMWH for testing
Heparini	No interference at therapeutic concentrations	-
DOAC	False (-) and false (+) LAC test are possible	Interrupt temporarily or use DOAC adsorption procedure
	**aCL and aβ2GPI (IgG and IgM)**	
Hemolysis/icterus/lipemia	Assay dependent	Check the reagent manufacturer’s instructions
Infection/inflammation	Transient (+) test possible	Retest after > 12 weeks
Rheumatoid factor IgM	False (+) aCL/aβ2GPI IgM	-
CRP - C-reactive protein. LAC - lupus anticoagulant. aPTT - activated partial thromboplastin time. DOAC - direct oral anticoagulant. aCL - anti cardiolipin antibodies. aβ2GPI - anti-β2-glycoprotein. LMWH - low-molecular-weight heparin.		

According to some researches, 60-70% of clinical decisions are influenced by the results of laboratory tests. In addition, at least 80% of evidence-based clinical guidelines aimed at diagnosing or treating patients have been shown to require laboratory testing (*62*). For laboratory analyses, the preanalytical phase of the determination is of great importance, such as the influence of the time of blood sampling. Luong *et al.* showed that in an apparently normal population, there are seasonal variations in the values of aCL (IgG and IgM) concentration. Values were lower in the summer months and higher in other months of the year. As higher values did not result in a thrombogenic effect, the authors hypothesized that these variations could be due to infections, which are rare in the summer months (*63*). When determining aβ2GPI and aCL, interference typical for immunochemical analyses, for example such as heterophile or human anti-animal antibodies, rheumatoid factor, high immunoglobulin concentrations, or factor inhibitors, should also be taken into account (*61*, *64*-*66*).

## Conclusion

Antiphospholipid syndrome is diagnosed based on clinical and laboratory criteria. The latter involve the determination of LAC, sbGPI and aCL on two occasions with an interval of 12 weeks. For patient safety, it is important to control all three phases of laboratory testing, *i.e.* preanalytical, analytical, and post-analytical phases. Specialists in laboratory medicine should take responsibility for the entire analytical process, so that possible interferences would be minimized, and doctors obtain reliable results of the patient’s laboratory findings in a timely manner. This will enable the doctor to establish a diagnosis and plan for further diagnostic and therapeutic procedures. Due to possible problems in performing tests on APL for a more reliable (optimal) interpretation of laboratory findings, a close cooperation between both, the laboratory specialists and the clinician specialists is needed.

## Data Availability

No data was generated during this study, so data sharing statement is not applicable to this article.
